# Association of HSD17B13 rs72613567: TA allelic variant with liver disease: review and meta-analysis

**DOI:** 10.1186/s12876-021-02067-y

**Published:** 2021-12-20

**Authors:** Shan Tang, Jing Zhang, Ting-Ting Mei, Wen-Yan Zhang, Su-Jun Zheng, Hai-Bin Yu

**Affiliations:** Beijing YouAn Hospital, Capital Medical University, 8 Xitoutiao, Youanmenwai Street, Beijing, 100069 China

**Keywords:** HSD17B13 rs72613567:TA allelic variant, Liver disease, Meta-analysis

## Abstract

**Background:**

To assess the association of HSD17B13 rs72613567:TA allelic variant with liver disease, we performed the current review and meta-analysis.

**Methods:**

Seven studies were identified by a search of CNKI,CBM,MEDLINE, PubMed, EMBASE, and CENTRAL databases from inception to November 2021. Odds ratios (ORs) with 95% confidence interval (CI) were calculated using random effects model or fixed effects model based on the between-study heterogeneity. The Stata 14.0 software was employed for data analysis.

**Results:**

Statistical analysis showed that the HSD17B13 rs72613567:TA allelic variant can decrease the risk of hepatocellular carcinoma(HCC) in nonalcoholic fatty liver disease (NAFLD) patients, alcoholic fatty liver disease (ALD) patients and viral hepatitis patients (TA vs T OR = 0.766, 95% CI = 0.682–0.860, P = 0.000; TATA + TAT vs TT OR = 0.755, 95% CI = 0.645–0.885, P = 0.001) or healthy controls(TA vs T OR = 0.649, 95% CI = 0.431–0.977, P = 0.038). Besides, the HSD17B13 rs72613567:TA allelic variant can also provide protection from nonalcoholic fatty liver disease (NAFLD) not only in entire population (TA vs T OR = 0.669, 95% CI = 0.524–0.856, P = 0.001) but also in healthy people (TA vs T OR = 0.600, 95% CI = 0.464–0.777, P = 0.000). No significant publication bias found in this airticle.

**Conclusion:**

The present findings suggest HSD17B13 rs72613567:TA allelic variant can reduce the risk of HCC and NAFLD in the entire population studied.

**Supplementary Information:**

The online version contains supplementary material available at 10.1186/s12876-021-02067-y.

## Background

Chronic liver disease (CLD) is a progressive deterioration of liver functions for more than six months with a broad spectrum of etiologies, including viral infection, nonalcoholic fatty liver disease (NAFLD), alcoholic fatty liver disease(ALD), autoimmune diseases, even hepatocellular carcinoma (HCC) [1]. CLD is a common and expensive condition, and studies of CLD-related hospitalizations have underestimated the true burden of disease [2]. CLD in the world is currently the fourth leading cause of death among persons aged 45 to 64 years [3]. Given significant advances in treatment of viral hepatitis, the burden of liver disease is shifting toward NAFLD [4]. The prevalence of NAFLD is estimated to be 10–40% in adults worldwide, and it is the most common liver disease in children and adolescents in developed countries [5]. HCC is life-threatening co-morbidities of NAFLD [6]. From 1999 to 2016 in the US annual deaths from HCC doubled to 11073 [7]. Therefore, assessing the genetic factors of HCC and NAFLD for early diagnosis or even treatment of the disease represents the key to reducing the high mortality rate. Nowadays, some studies have shown that hydroxysteroid 17-β dehydrogenase family 13 (HSD17B13) rs72613567:TA allelic variant is associated with liver disease [8]. Previous studies investigating the association between the HSD17B13 rs72613567: TA allelic variant and liver disease have given controversial results due to differences in population samples, detection methods and diagnostic criteria [9]. In order to unify these differences, a meta-analysis of published research was conducted to comprehensively assess the relationship between HSD17B13 rs72613567: TA allelic variant and liver disease.

## Methods

The current meta-analysis complied with Preferred Reporting Items for Systematic Reviews and Meta-Analyses (PRISMA) guidelines (PROSPERO CRD42020178246) [10].

### Search strategy

Relevant publications were identified through searching PubMed (Medline), China National Knowledge Infrastructure (CNKI), CBM, EMBASE and CENTRAL web databases using the following search terms: “Hydroxysteroid 17-β dehydrogenase 13” and “Liver Diseases”. What is more, we also checked the references of relevant articles to find any other potentially relevant papers. Table 1 summarizes the search strategy for PubMed, and it was also employed for all databases. The last literature search in the above databases was completed on November 20, 2021.

**Table 1:** PubMed search strategy

Number	Search items
#1	"Hydroxysteroid 17-β dehydrogenase 13" OR "Hydroxysteroid 17-BETA dehydrogenase 13" OR "17-beta-hydroxysteroid dehydrogenase 13" OR "17-β-hydroxysteroid dehydrogenase 13" OR HSD17B13 OR rs72613567 OR rs6834314 OR rs62305723
#2	"Liver Diseases"[Mesh] OR Liver* OR hepatoma* OR Intrahepatic* OR Hepatic* OR Hepatis* OR hepatocellular* OR hepatitis* OR cirrhosis* OR HBV OR HCV OR HCC OR fibrosis* OR ALD OR NAFLD OR Hepatolenticular* OR Hepatomegaly* OR Hepatopulmonary* OR Hepatorenal*
#3	#1 AND #2

### Inclusion and exclusion criteria of the literature

Inclusion criteria: (1) involving the associations between HSD17B13 rs72613567: TA allelic variant and liver disease; (2) case–control studies or cohort studies; (3) if two (or more) studies included the same population, the most recent was included to avoid repeated statistics; (4) providing complete data on genotype frequencies. (5) the diagnosis criteria of HCC or NAFLD is explained in the study. (6) no restrictions on language and age of participants. Exclusion criteria: (1) no clear diagnostic criteria for liver disease described; (2) not reported the genotype frequencies; (3) the OR values and 95% CI were not available by calculation; (4) case reports; (5) in vitro or animal studies.

### Data extraction

The information extracted by two independent investigators from each study included:the first author's surname, publication year, country in which the study was conducted, total numbers of patients in the case and control groups, sex ratio, as well as the numbers of cases and controls with the TA and T genotypes, whether genotype distribution was consistent with the Hardy–Weinberg equilibrium (HWE). Any disagreement was resolved by discussing with the third author until we reached a group consensus.

### Statistical analysis

All the statistical analyses were performed with stata 14.0 software (StataCorp LLC, College Station, TX, USA), with probability value of < 0.05 considered to be statistically signifcant.Summary odds ratio (ORs) and 95% confidence intervals (CIs) were calculated to evaluate the association of the TA/T polymorphism in the HSD17B13 gene with liver disease under allele genetic contrast. Heterogeneity was measured by Cochran’s Chi square-based Q test and *I*^*2*^ -statistic for all qualifed studies. If *P* < 0.05 or *I*^*2*^ > 50%, indicating significant heterogeneity, the random-effects model was applied to calculate pooled ORs. otherwise, the fixed-effects model was utilized. The suitability test was used to check whether the gene distribution of the control group consistent with the Hardy–Weinberg equilibrium, and the PH-W > 0.05 was regarded as consistent with the HWE. We also performed sensitivity analysis to explore the effect of a single study on overall results by removing one study sequentially. Egger's regression asymmetry test, Begger's regression asymmetry test and funnel plot were employed to assess publication bias.

## Results

### Study characteristics

**Fig 1:**
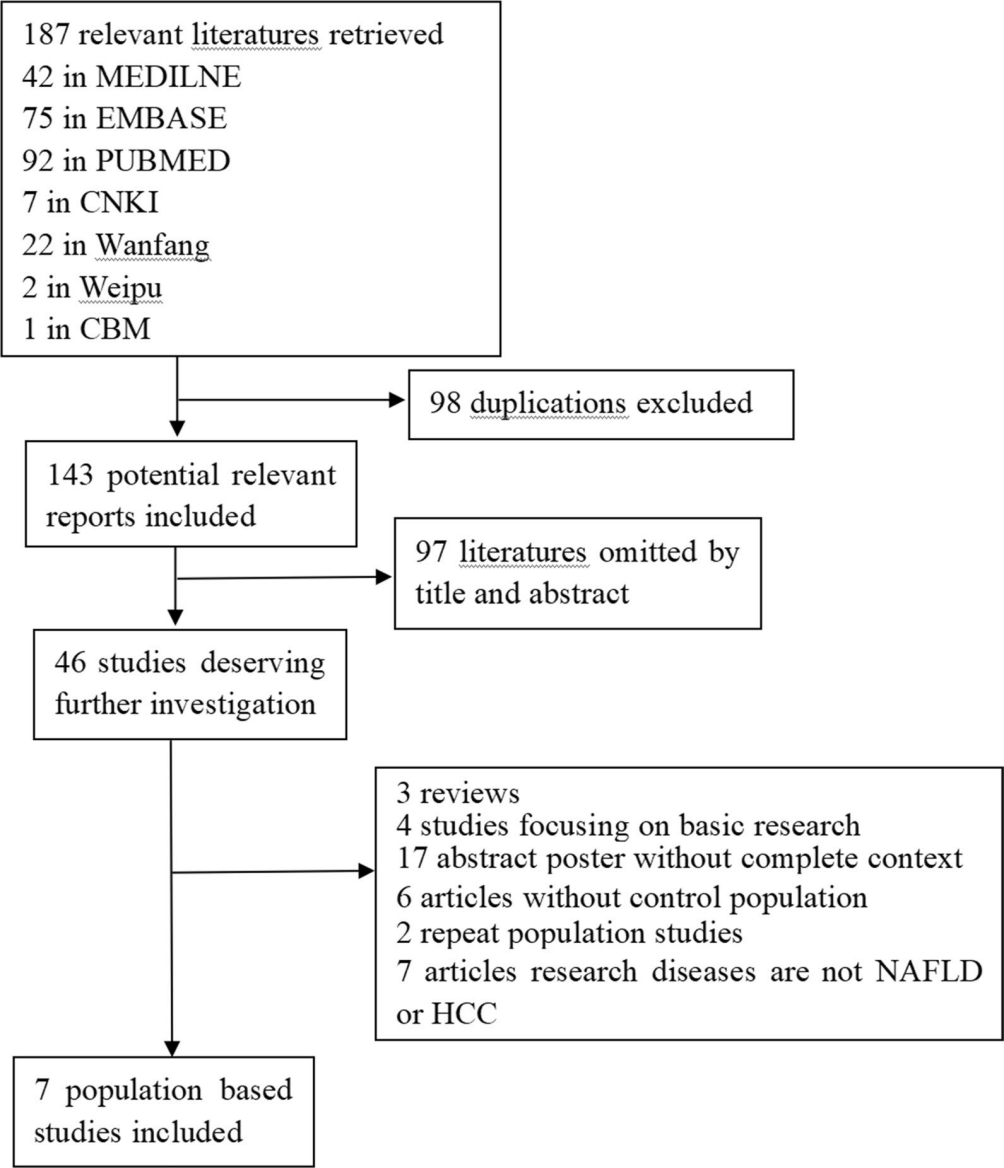
Flow diagram for study selection

Figure 1 depicted the fow diagram of literature research and selection. Initially, we identifed 187 relevant studies in the databases before November 2021.After exclusion of duplicate or irrelevant to this meta-analysis, 46 remaining literature seemed to be eligible for this meta-analysis. The second-round of review was based on careful full-text review of the 46 retained papers. Then, 39 reports were excluded as follows: reviews (n = 3), focusing on basic research (n = 4), abstract poster (n = 17), without control population (n = 6), repeat population (n = 2), research diseases are not NAFLD or HCC (n = 7).Eventually, this meta-analysis included seven case–control studies.A total of patients with 1384 liver cancer, 2990 patients with chronic liver disease (NAFLD, ALD, viral hepatitis), 4621 healthy controls, 1104 patients with NAFLD and 5225 individuals without NAFLD were included. The main characteristics and genotyping data for all studies are summarized in Table 2.

**Table 2:** Characteristics of the studies included in the meta-analysis

Study	Cohort characteristics	Country	Total numbers	Gender, (men%)	Age (mean ± SD)	Study design	Genotyping	Allele		HWE	Risk factors	Matching variables
								TA	T			
Stickel [11]	HCC	Swiss, German, Austrian, Italian, British	1031	91.0	62 ± 10	Case control study	TaqMan	365	1697	YES	Alcohol consumption	Age, gender, BMI, PNPLA3 (rs738409)
	ALD		1653	84.0	48 ± 10			728	2578			
Yang [12]	HCC	France	178	83.0	64	Case control study	TaqMan	86	270	YES	Alcohol consumption	Age, gender, PNPLA3 (rs738409)
	Viral hepatitis		459	68.0	55			316	602			
Fazio [13]	HCC	Italy	123	–	–	Case control study	TaqMan	52	194	–	NO	PNPLA3 (rs738409)
	NAFLD		241		–			98	384			
Abul-Husn [14]	HCC	USA	44	42.0	63	Case control study	TaqMan	12	76	YES	Alcohol consumption	Age, gender, BMI
	NAFLD		435					106	764			
Scheiner [15]	HCC	Vienna	23	–	–	Case control study	TaqMan	10	40	NO	Alcohol consumption	Age, gender, MELD, HVPG
	Viral hepatitis and NAFLD		464	74.0	–			204	720			
Abul-Husn [14]	HCC	USA	44	42.0	63	Case control study	TaqMan	12	76	YES	Alcohol consumption	Age, gender, BMI
	Healthy controls		4279					1172	7386			
Yang [12]	HCC	France	1018	83.0	–	case control study	TaqMan	692	1344	YES	alcohol consumption	Age, gender, PNPLA3 (rs738409)
	Healthy controls		503	–	–			402	604			
Fazio [13]	HCC	Italy	123	–	–	Case control study	TaqMan	52	194	–	NO	PNPLA3 (rs738409)
	Healthy controls		90		–			50	130			
Ting [16]	NAFLD	Malaya, China, India	223	52.5	55.65 ± 12.5	Case control study	TaqMan	107	339	–	NO	Age, gender, BMI, Blood pressure,
	Viral hepatitis		205	42.4	51.0 ± 12.5			139	271			
Di Sessa [17]	NAFLD	Italy	318	53.3	10.7 ± 2.9	Case control study	TaqMan	200	436	YES	NO	Age, gender, BMI,
	Healthy controls		366	50.5	10.3 ± 2.3			334	398			
Fazio [13]	NAFLD	Italy	151	–	–	Case control study	TaqMan	48	254	-	NO	PNPLA3 (rs738409)
	Healthy controls		90		–			50	130			
Abul-Husn [14]	NAFLD	USA	210	42.0	63	Case control study	TaqMan	46	374	YES	alcohol consumption	Age, gender, BMI
	Healthy controls		4279					1172	7386			
Scheiner [15]	NAFLD	Vienna	202	76.0	59 ± 10	Case control study	TaqMan	89	315	NO	NO	Age, gender, MELD, HVPG
	Viral hepatitis		285	74.0	52 ± 9			125	445			

### Meta-analysis results

#### Association between HSD17B13 rs72613567: TA allelic variant and HCC

**Fig 2:**
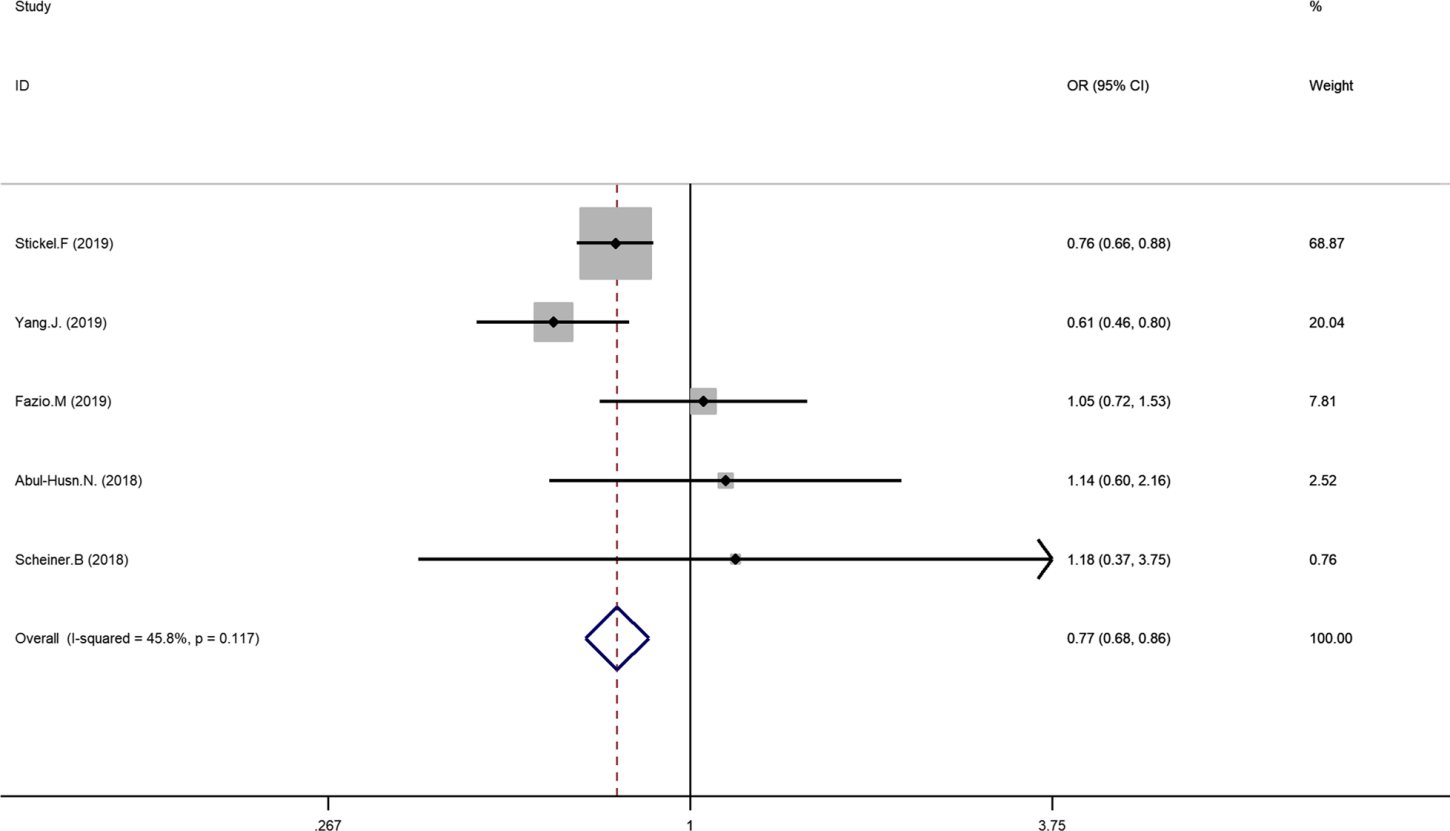
Forest plot of studies evaluating the OR with 95%CI of HSD17B13 rs72613567:TA allelic varient in HCC patients compared with chronic liver disease

**Fig 3:**
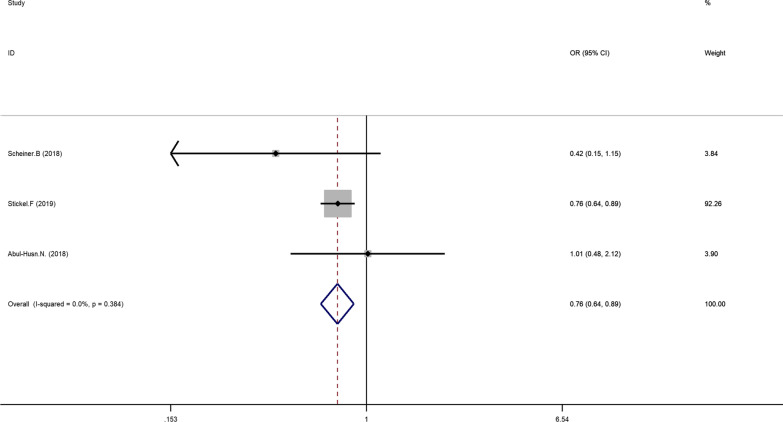
Forest plot of studies evaluating the OR with 95%CI of HSD17B13 rs72613567:TA allelic varient in HCC patients compared with chronic liver disease

**Fig 4:**
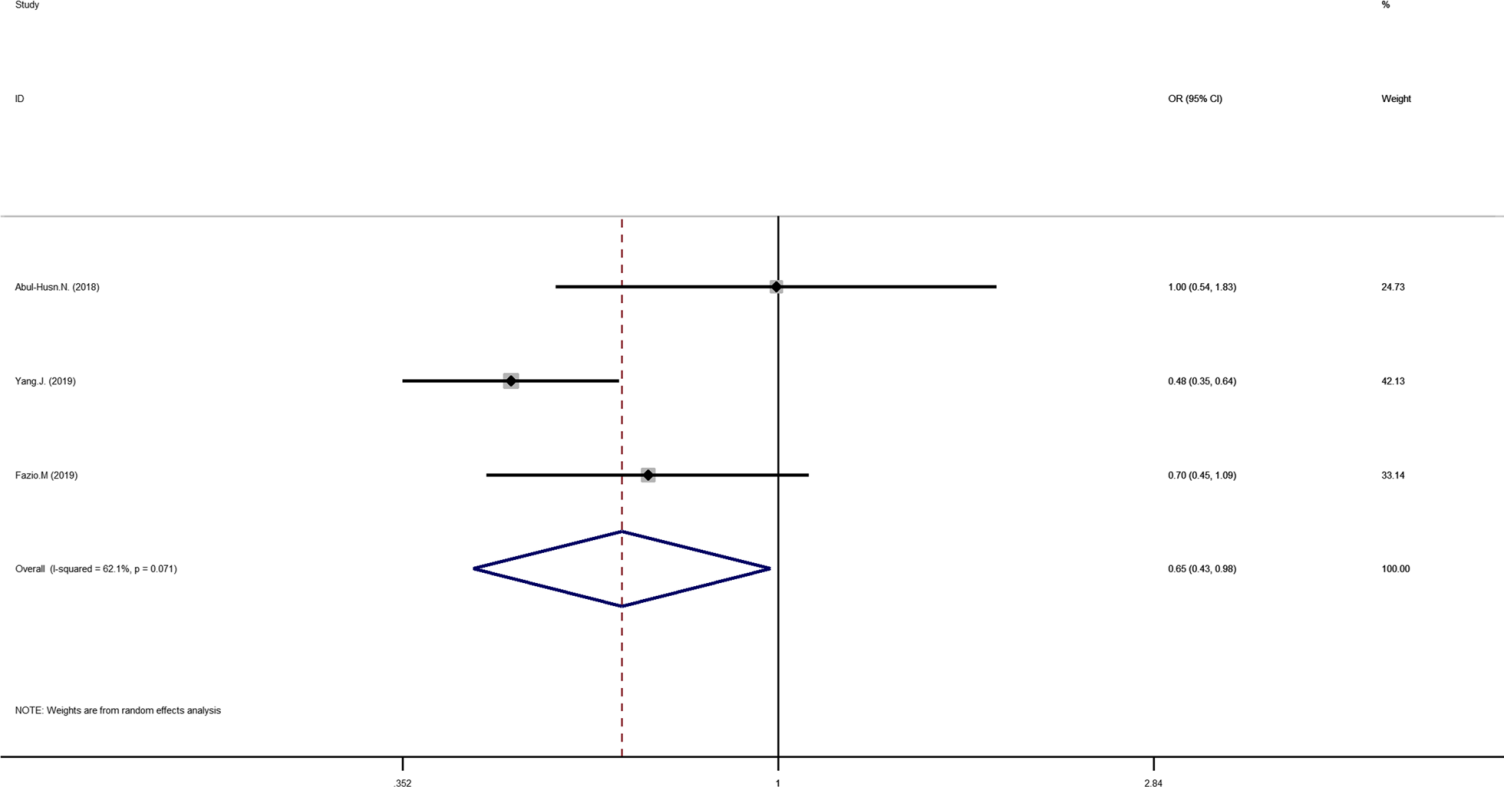
Forest plot of studies evaluating the OR with 95%CI of HSD17B13 rs72613567:TA allelic varient in HCC patients compared with healthy controls

The main results of the meta-analysis are displayed in Table 3. Five literatures described the association between HSD17B13 rs72613567: TA allelic variant and susceptibility to hepatocellular carcinoma (HCC) compared with chronic liver disease (NAFLD, ALD, viral hepatitis). Using fixed-effect model, we found that patients with HSD17B13 rs72613567: TA allelic variant are less likely to develop liver cancer under allelic model: TA vs T OR = 0.766, 95% CI = 0.682–0.860, P = 0.000 (Fig. 2). The additive model can be analyzed in three literatures. The results showed that the risk of liver cancer in the TATA + TAT genotype group was lower than that of the TT genotype group (TATA + TAT vs TT OR = 0.755, 95% CI = 0.645–0.885, P = 0.001; Fig. 3). Besides, this meta-analysis involved three articles on the association between HSD17B13 rs72613567: TA allelic variant and susceptibility to HCC compared with healthy controls. The random effects model was employed for pooled ORs since significant heterogeneity was detected. We discovered that HSD17B13 rs72613567: TA allelic variant has protective effect on liver cancer: TA vs T OR = 0.649, 95% CI = 0.431–0.977, P = 0.038 (Fig. 4).

**Table 3:** Meta-analysis of the association of HSD17B13 rs72613567: TA allelic variant and liver disease susceptibility

Liver disease		Number of studies		Relevance test		Heterogeneity test		Publication	
		OR(95% CI)		Z	P_value_	I^2^	Q	P_hel_	P_egger_
HCC compared with CLD (allelic model)	5	0.766(0.682–0.860)	4.51	0.000	45.8	7.38	0.117	0.378	1.03
HCC compared with CLD (additive model)	3	0.755(0.645–0.885)	3.47	0.001	0	1.91	0.384	0.845	-0.25
HCC compared with healthy controls (allelic model)	3	0.648(0.429–0.979)	2.06	0.039	62.1	5.28	0.071	0.033	19.01
NAFLD compared with NON-NAFLD (allelic model)	5	0.669(0.524–0.856)	3.20	0.001	68.9	12.87	0.012	0.280	0.800
NAFLD compared with healthy controls (allelic model)	3	0.600(0.464–0.777)	3.89	0.000	50.3	4.02	0.134	0.060	0.963

#### Association between HSD17B13 rs72613567: TA allelic variant and NAFLD

**Fig 5:**
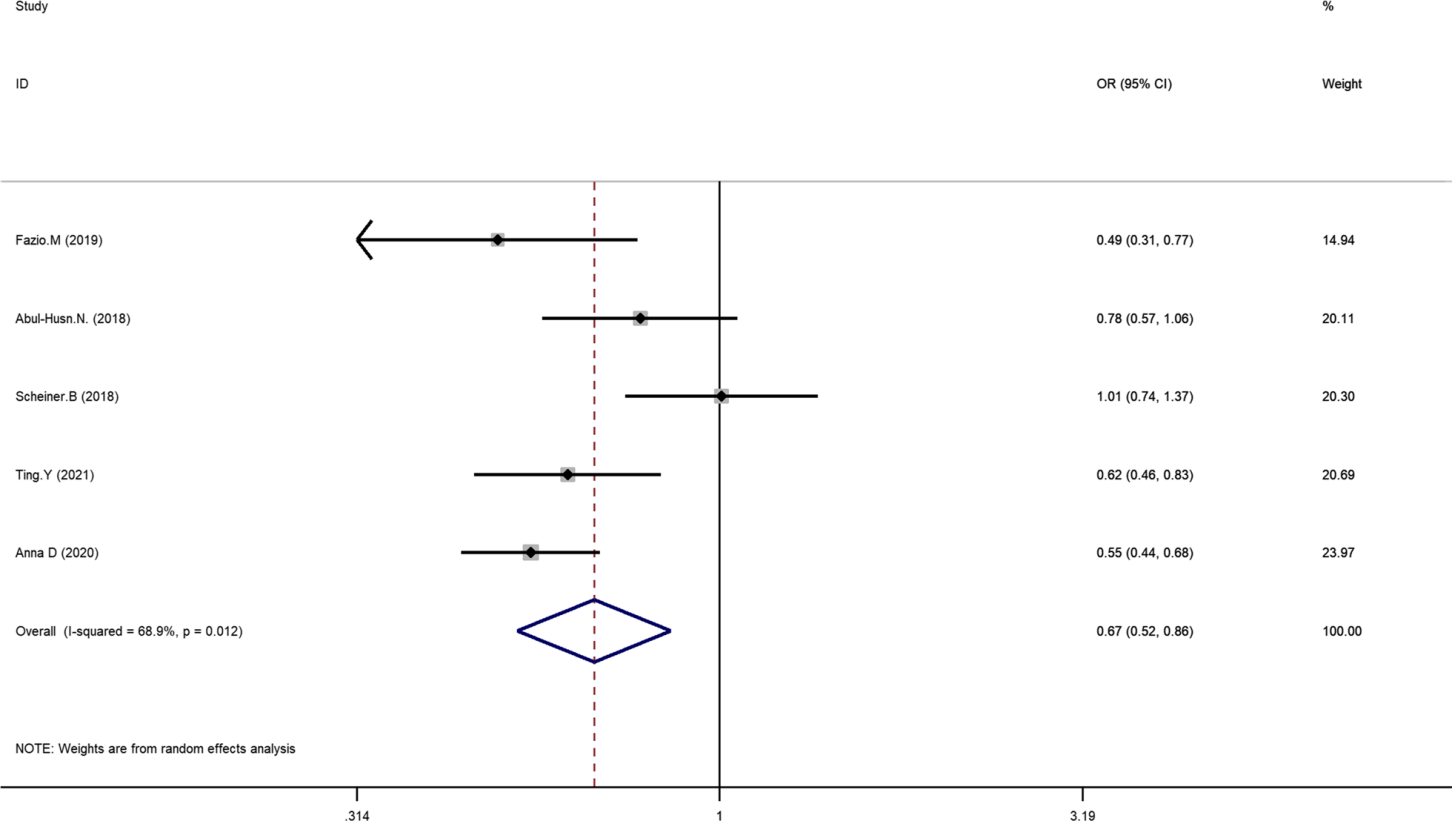
Forest plot of studies evaluating the OR with 95%CI of HSD17B13 rs72613567:TA allelic varient in NAFLD compared with non-NAFLD

**Fig 6:**
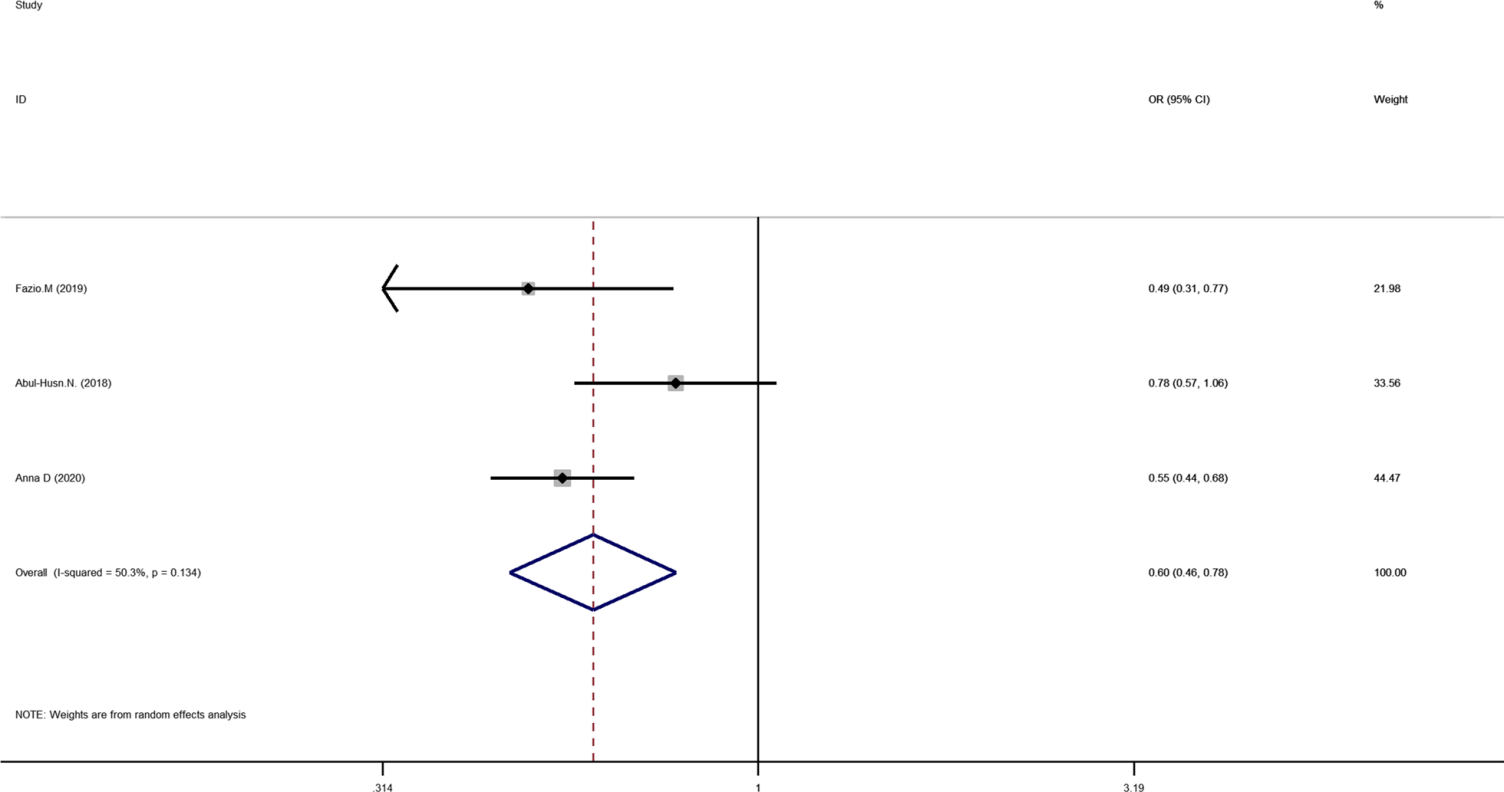
Forest plot of studies evaluating the OR with 95%CI of HSD17B13 rs72613567:TA allelic varient in NAFLD compared with healthy controls.

Five articles were included in the HSD17B13 rs72613567: TA allelic variant and risk of NAFLD. Using random-effect model, we found the HSD17B13 rs72613567: TA allelic variant can reduce the risk of NAFLD in the entire population: TA vs T OR = 0.669, 95% CI = 0.524–0.856, P = 0.001 (Fig. 5). To evaluate the impact of the HSD17B13 rs72613567 variant on the risk of NAFLD in healthy controls, we performed a meta-analysis involved three articles. The results showed that the risk of NAFLD in the HSD17B13 rs72613567: TA allelic variant group was lower than that of individuals without the TA allelic variant (TA vs T OR = 0.600, 95% CI = 0.464–0.777, P = 0.000; Fig. 6).

### Sensitivity analysis and publication bias

In the meta-analysis,the results of sensitivity analysis showed that no single study impacted the overall ORs qualitatively, confirming the statistical stability of our findings(Additional file 1: Figs. 1–5).

In the meta-analysis of HSD17B13 rs72613567: TA allelic variant and liver disease susceptibility, the Egger regression analysis suggested that obviously publication bias existed in HCC patients compared with healthy controls. (Additional file 1: Fig. 12). Examination of the pruning method showed that the funnel chart was basically symmetrical after pruning, and the OR difference in the combined effect before pruning was statistically significant (OR = 0.648, 95% CI = 0.429–0.979, P = 0.039). The OR difference in the combined effects after trimming was also statistically significant (OR = 0.476, 95% CI = 0.312—0.727, P = 0.001), indicating that there was no significant publication offset after trimming, and the results were robust (Additional file 1: Fig. 13). And no publication bias existed in other groups (Additional file 1: Figs. 6–19).

## Discussion

The human HSD17B13 gene is located on chromosome 4 and contains 7 exons [18]. HSD17B13 gene codes for a retinol dehydrogenase, 17β-hydroxysteroid dehydrogenase type 13, which is highly expressed in the liver [19]. HSD17B13 is a member of the short-chain dehydrogenase/reductase family [20]. Although the function of HSD17B13 is still unclear yet, it appears to be involved in the metabolism of steroid hormones, prostaglandins, lipids, xenobiotics, and retinoids [21]. There are two main types of HSD17B13 gene mutations associated with liver disease including rs72613567 and rs6834314. And this two mutations have strong linkage disequilibrium. The rs6834314 A > G is located 11 kb downstream of HSD17B13, and is associate with NAFLD [18]. The rs72613567 mentioned in this meta-analysis is an indel in a non-coding region between exon 6 and exon 7 of the HSD17B13 gene, of which the predicted functional consequence is a splice donor variant of HSD17B13 gene, which can produce unstable protein and reduce the activity of HSD17B13 enzyme [22].

Although the research on the relationship between HSD17B13 rs72613567: TA allelic variant and liver disease has attracted the attention of many researchers, the conclusion is not clear. In the study of the relationship between the HSD17B13 gene and the risk of HCC, Yang et al. [23] found that compared with healthy people, the frequency of HSD17B13 rs72613567: TA allele in HCC patients was significantly reduced. Among ALD patients, the proportion of HCC patients carrying TA alleles was significantly lower than that patients without HCC, indicated that HSD17B13 rs72613567: TA allelic variant may play a protective role in the development of HCC in ALD patients. At present, the mechanism of the protective effect of HSD17B13 rs72613567 on HCC is not clear, but Chen et al. found that HSD17B13 induced an accumulation of the Huh-7 and SK-HEP-1 hepatoma cell lines in G1 phase and reduction of cells in S and G2 phase, indicated that overexpression of HSD17B13 can delay the cell cycle G1/S progression [24]. In this meta-analysis, we found that the HSD17B13 rs72613567:TA allelic variant can decrease the risk of HCC in CLD (TA vs T OR = 0.766, 95% CI = 0.682–0.860, P = 0.000; TATA + TAT vs TT OR = 0.755, 95% CI = 0.645–0.885, P = 0.001) or healthy controls(TA vs T OR = 0.649, 95% CI = 0.431–0.977, P = 0.038). This is consistent with previous research results.

In the study of the relationship between the HSD17B13 gene and the risk of NAFLD, the results vary from study to study. Su et al. [25] found that HSD17B13 was highly expressed in NAFLD patients. In 2019, Ma et al. also reached the same conclusion, which indicates that HSD17B13 may be a risk factor for the onset of NAFLD [18]. However, Adam et al. [26] came to the opposite conclusion, they found the expression of key fatty acid synthesis proteins in the liver of HSD17B13 knockout mice increased, and these mice are more likely to develop severe NAFLD. The differences in species could be responsible for the contradictory results. The mechanism of how the HSD17B13 genetic variant effect the NAFLD has yet to be clarified in detail [27]. In this meta-analysis, we found that the HSD17B13 rs72613567:TA allelic variant can provide protection from NAFLD not only in entire population (TA vs T OR = 0.669, 95% CI = 0.524–0.856, P = 0.001) but also in healthy people (TA vs T OR = 0.600, 95% CI = 0.464–0.777, P = 0.000).

In addition to research on HCC and NAFLD in this meta-analysis, Peter et al. [28] determined HSD17B13:TA (rs72613567) variant by allelic discrimination real-time PCR in 586 Wilson’s disease (WD) patients. They get the conclusion that the HSD17B13: TA allele modulates the phenotype and outcome of WD. This allele likely ameliorates hepatic fibrosis and reduces the transition from copper induced hemolysis to fulminant disease in patients with WD.

In this study, we developed a retrieval strategy and conducted literature quality evaluation according to the requirements of the Oxford Critical Appraisal Skill Program (Oxford CASP, 2004), and use suitable mathematical models to perform quantitative analysis of multiple identical or similar research results, increasing the test efficiency of research results.

To our knowledge, this is the first meta-analysis to analyze the effects of HSD17B13 rs72613567: TA allelic variant on the risk of NAFLD in healthy population and viral hepatitis patients. And for the first time, the result was obtained that HSD17B13 rs72613567 can reduce the risk of NAFLD. Besides, we not only analysis the association between HSD17B13 rs72613567: TA allelic variant and susceptibility to hepatocellular carcinoma (HCC) compared with healthy controls, but also compared with CLD. That means that HSD17B13 rs72613567 can not only reduce the risk of HCC in healthy people, but also have a protective effect in patients with CLD.

Currently, there is no specific treatment for HCC and NAFLD. The results got in the meta-analysis demonstrated that HSD17B13 rs72613567 can protect against HCC and NAFLD. This could be the light for seeking a therapeutic target for HCC and NAFLD. Along with the advance of genomic studies, genome editing techniques have also tremendously developed. Using Gene editing techniques such as transcriptional activator-like effector nuclease (TALEN) and CRISPR-associated nuclease [29], the HSD17B13 genotype could be genetically edited in hepatocytes in the future.

Meanwhile, there are still some limitations in this article. Firstly, due to the lack of a unified document quality evaluation standard, the included articles are subjectively selected and evaluated, which may affect the stability of the meta-analysis results. Secondly, since meta-analysis itself is a retrospective study, there is a degree of bias. Thirdly, due to the limited number of articles included, the credibility of the results of the meta-analysis may be impacted. Fourthly, this study only included case control studies, which may affect the credibility of the results. Fifthly, obesity, age, gender, alcohol intake, intake of the fungal metabolite aflatoxin, and hepatitis B and C infections may contribute to HCC and NAFLD, but we did not take these into consideration. Due to these limitations, we still need to expand the sample size to further and systematically evaluate studies.

In summary, the polymorphism of HSD17B13 rs72613567: TA allelic variant can reduce the risk of HCC and NAFLD.

## Conclusions

The present findings suggest HSD17B13 rs72613567:TA allelic variant can reduce the risk of hepatocellular carcinoma and NAFLD in the entire population studied.

## Supplementary Information

**Additional file 1.** Sensitivity analysis and bias analysis figures.

## Data Availability

All data generated or analyzed during this study are derived from previously published original research articles. Details are available from the corresponding author on reasonable request.

## Abbreviations

CLD: Chronic liver disease
ALD: Alcoholic liver disease
NAFLD: Nonalcoholic fatty liver disease
HCC: Hepatocellular carcinoma
PRISMA: Preferred reporting items for systematic reviews and meta-analyses
CNKI: China national knowledge infrastructure
ORs: Odds ratio
CIs: Confidence intervals
WD: Wilson disease
