# Supplementary material for: Association of HSD17B13 rs72613567: TA allelic variant with liver disease: review and meta-analysis
Source: BMC Gastroenterol. 2021 Dec 20;21:490. doi: 10.1186/s12876-021-02067-y (PMC8686634; doi:10.1186/s12876-021-02067-y)
Supplement: Supplementary file 1 — Additional file 1. Sensitivity analysis and bias analysis figures. [file 12876_2021_2067_MOESM1_ESM.docx]

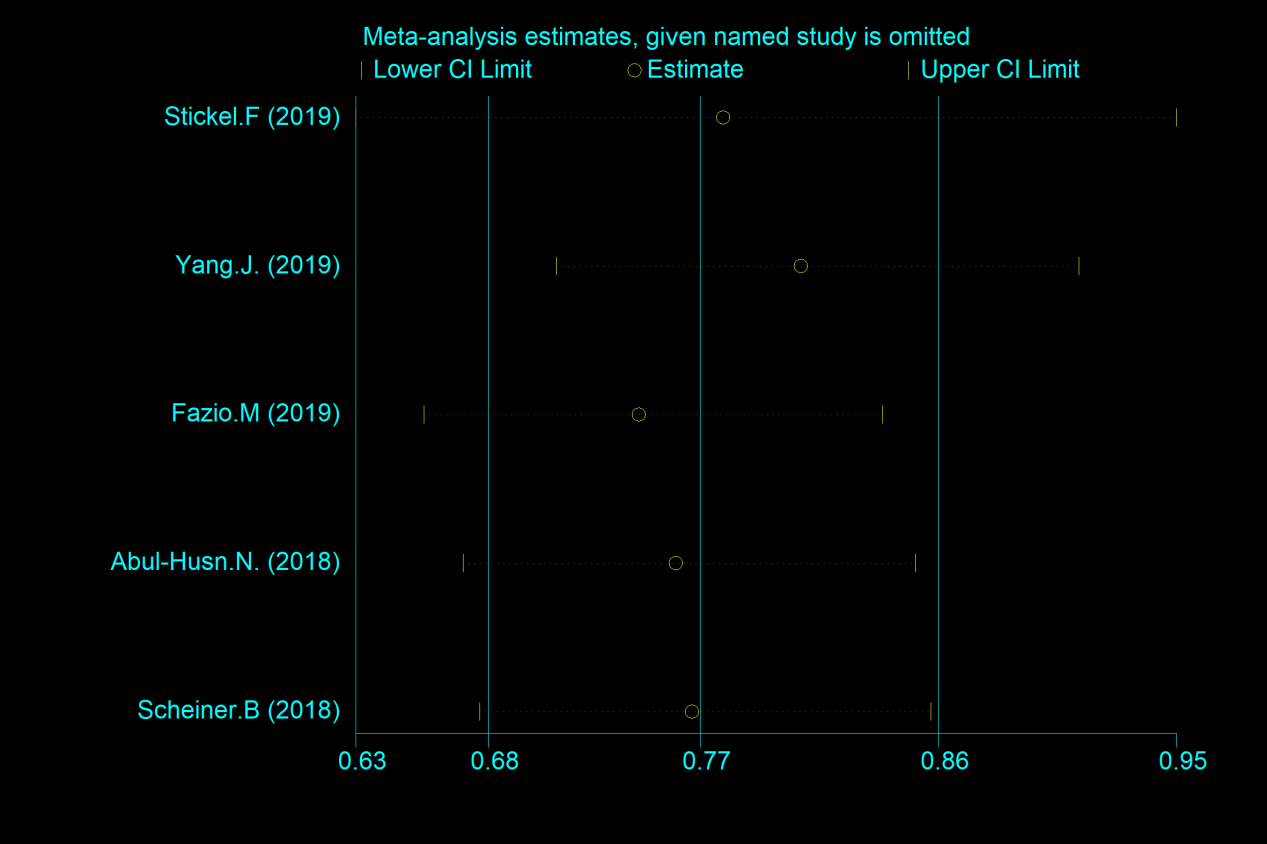


Supplementary Figure 1 Sensitivity analysis of HSD17B13 rs72613567: TA allelic variant in HCC patients compared with chronic liver disease (allelic model).


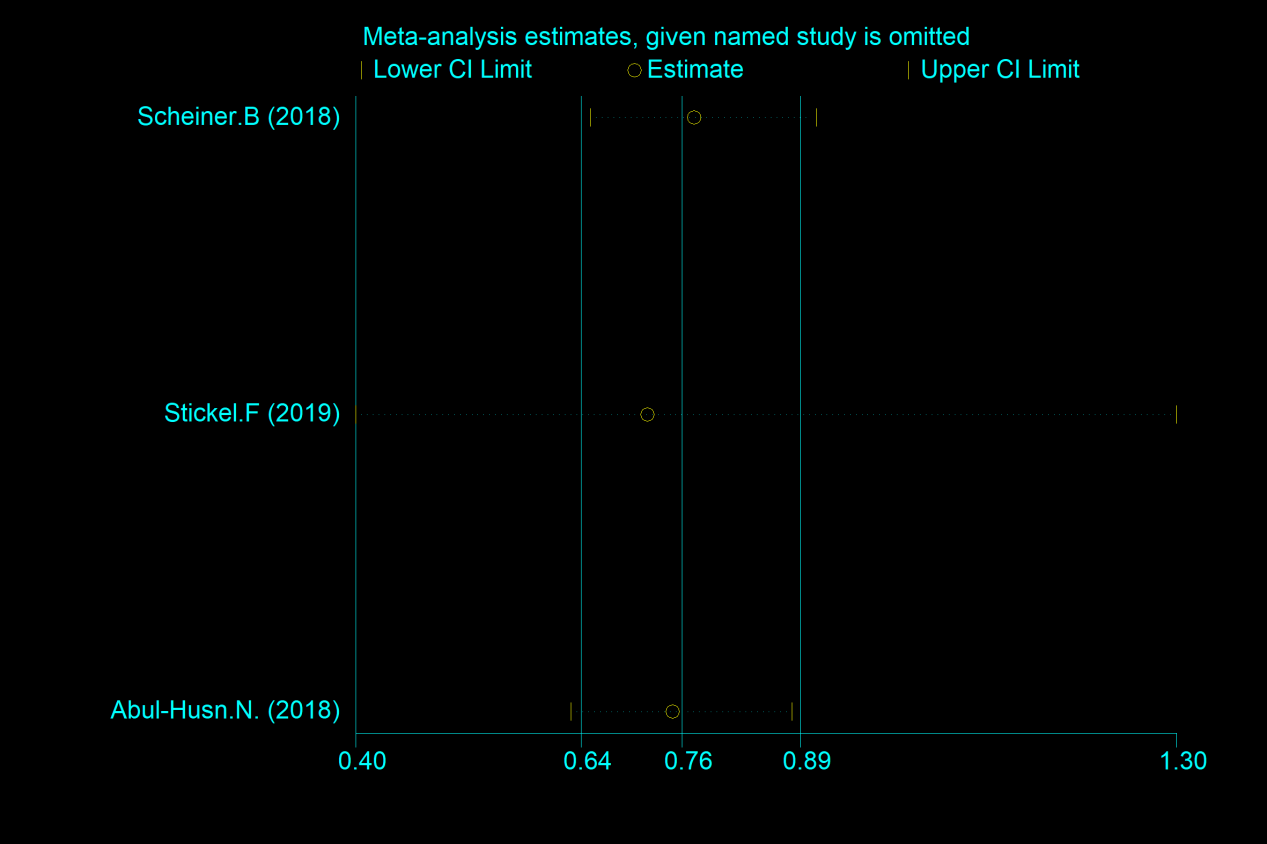


Supplementary Figure 2 Sensitivity analysis of HSD17B13 rs72613567: TA allelic variant in HCC patients compared with chronic liver disease (additive model).


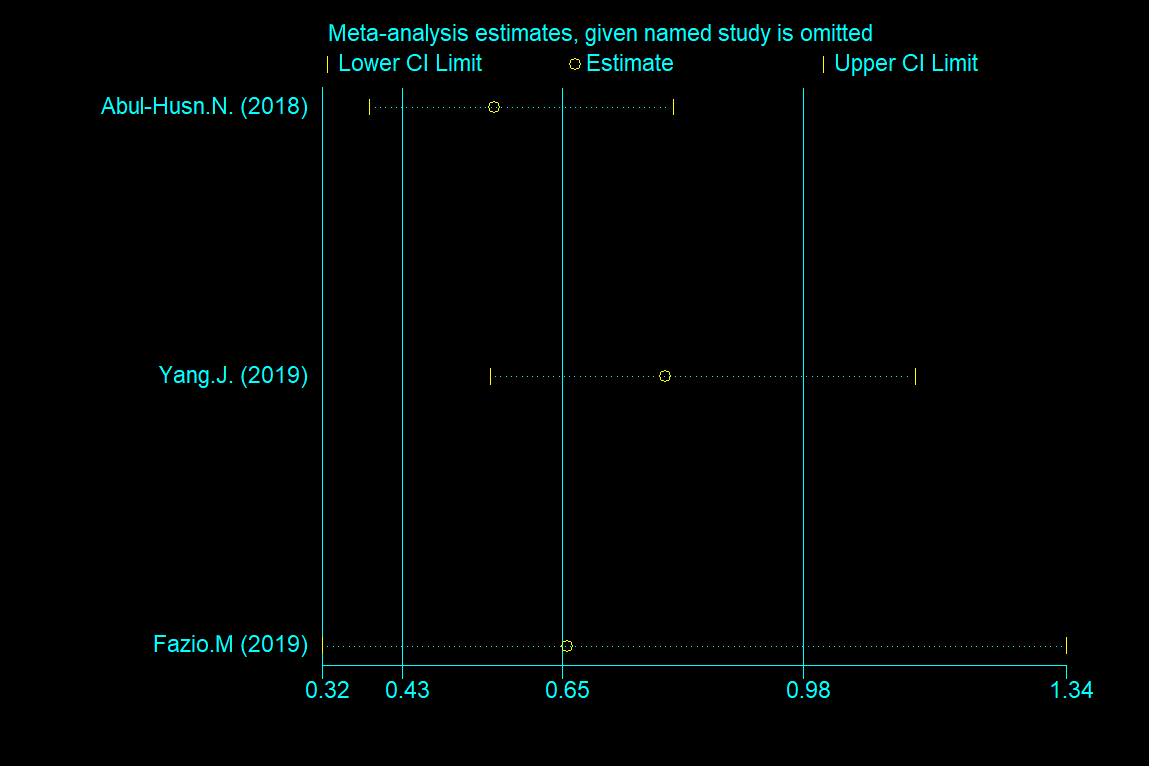


Supplementary Figure 3 Sensitivity analysis of HSD17B13 rs72613567: TA allelic variant in HCC patients compared with healthy controls.


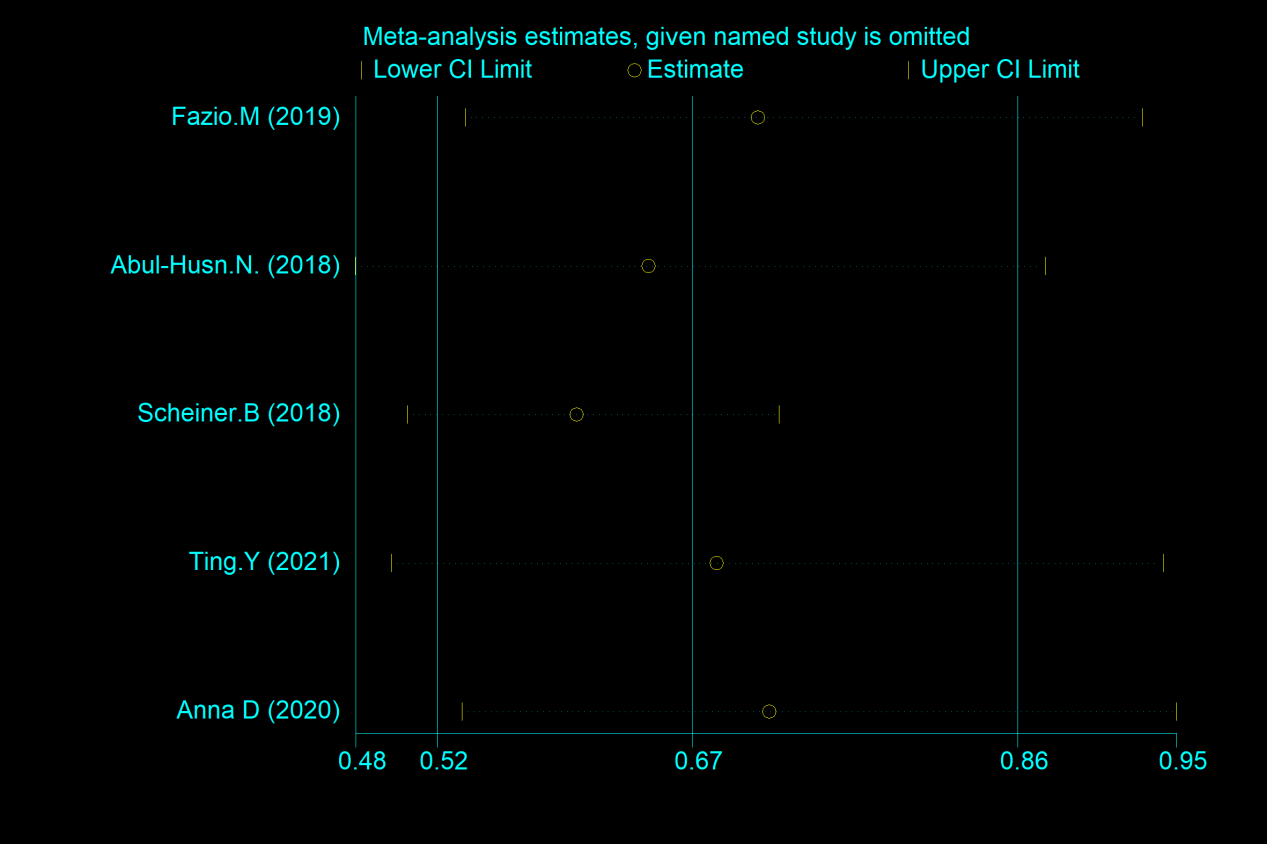


Supplementary Figure 4 Sensitivity analysis of HSD17B13 rs72613567: TA allelic variant in NAFLD patients compared with non-NAFLD(healthy controls and viral hepatitis).


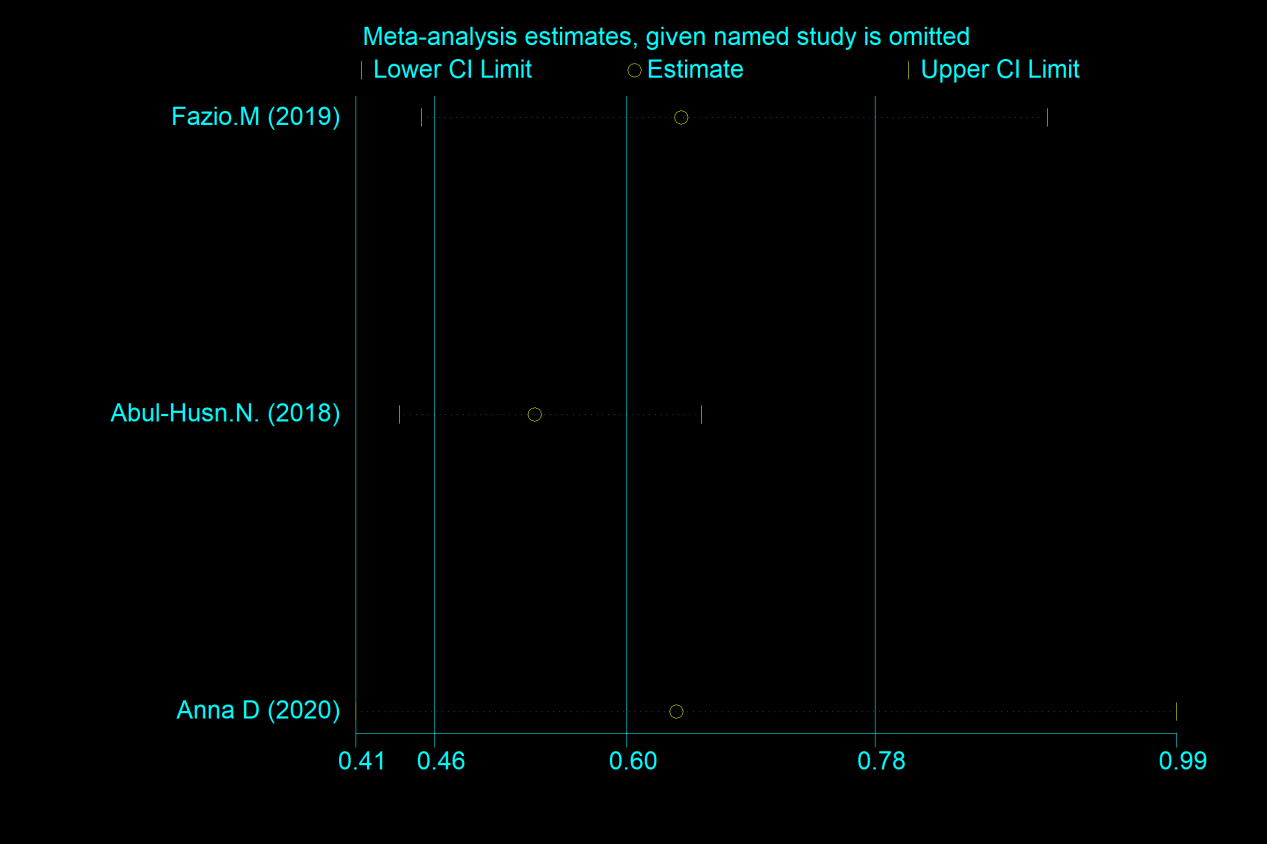


Supplementary Figure 5 Sensitivity analysis of HSD17B13 rs72613567: TA allelic variant in NAFLD patients compared with healthy controls.


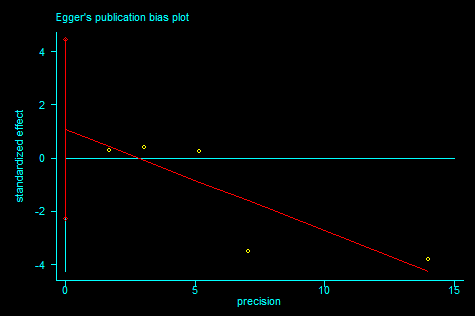


Supplementary Figure 6 Egger's funnel plot of HSD17B13 rs72613567: TA allelic variant in HCC patients compared with chronic liver disease (allelic model).


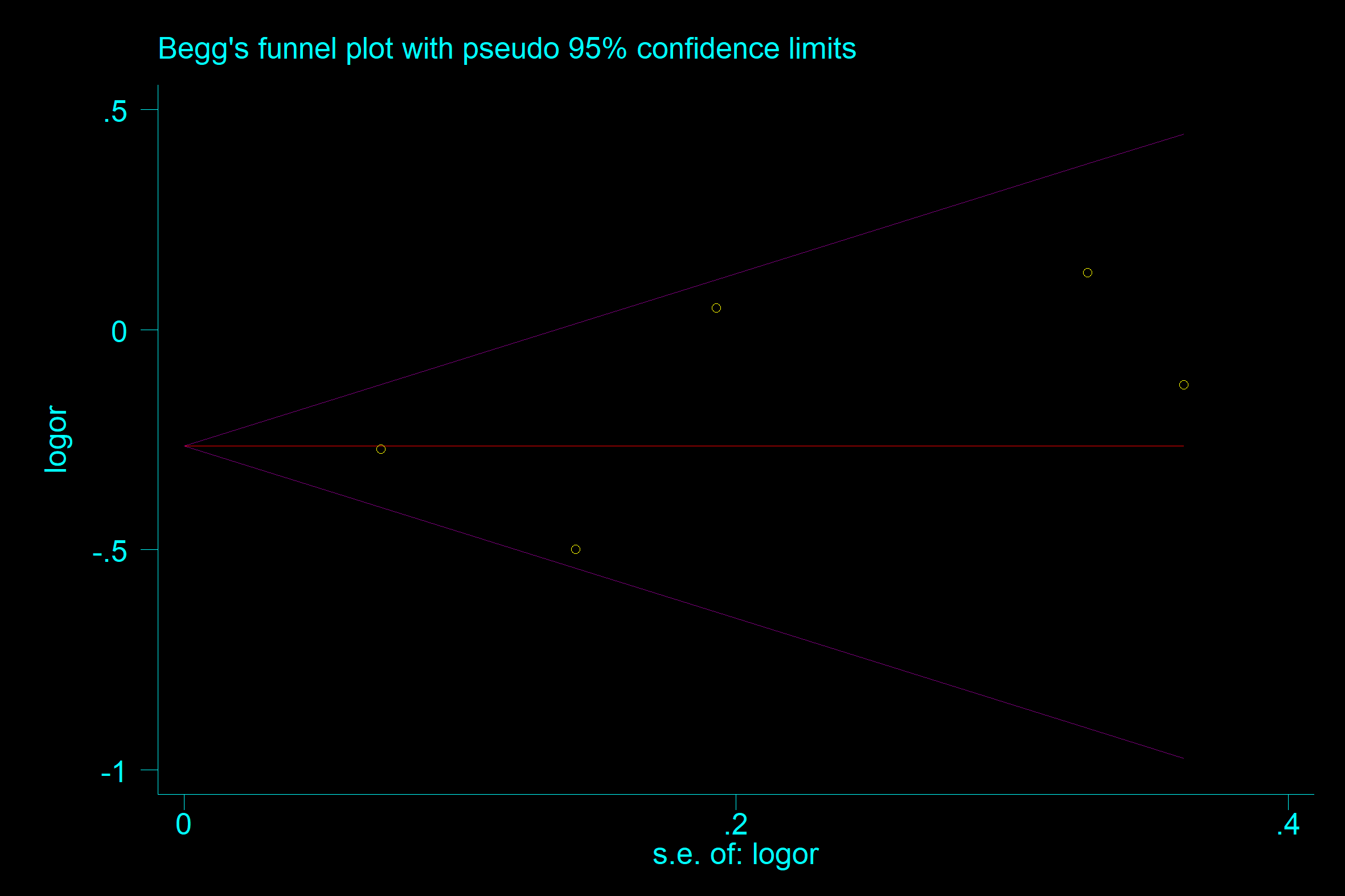


Supplementary Figure 7 Begger's funnel plot of HSD17B13 rs72613567: TA allelic variant in HCC patients compared with chronic liver disease (allelic model).


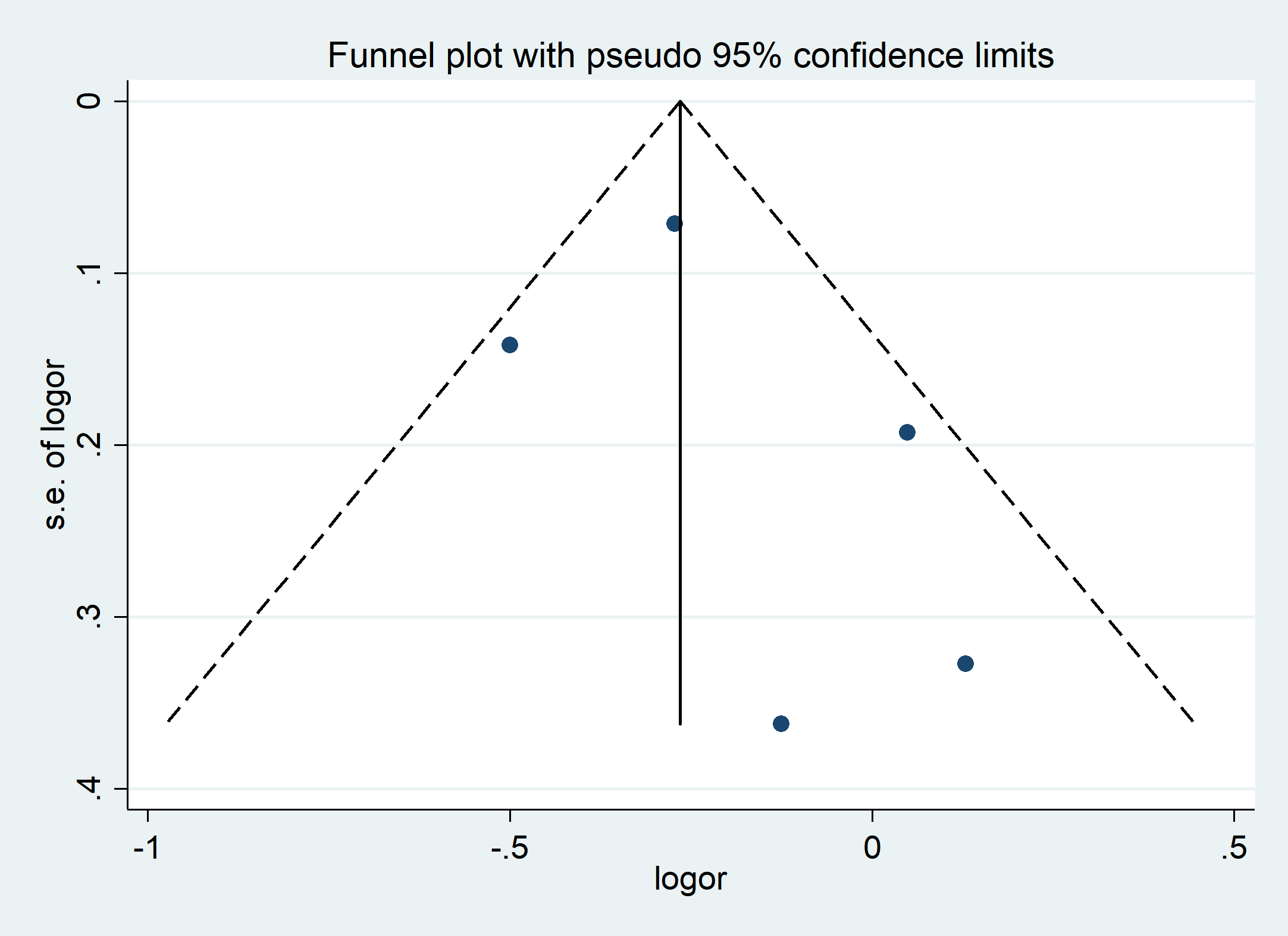


Supplementary Figure 8 Funnel plot of HSD17B13 rs72613567: TA allelic variant in HCC patients compared with chronic liver disease (allelic model).


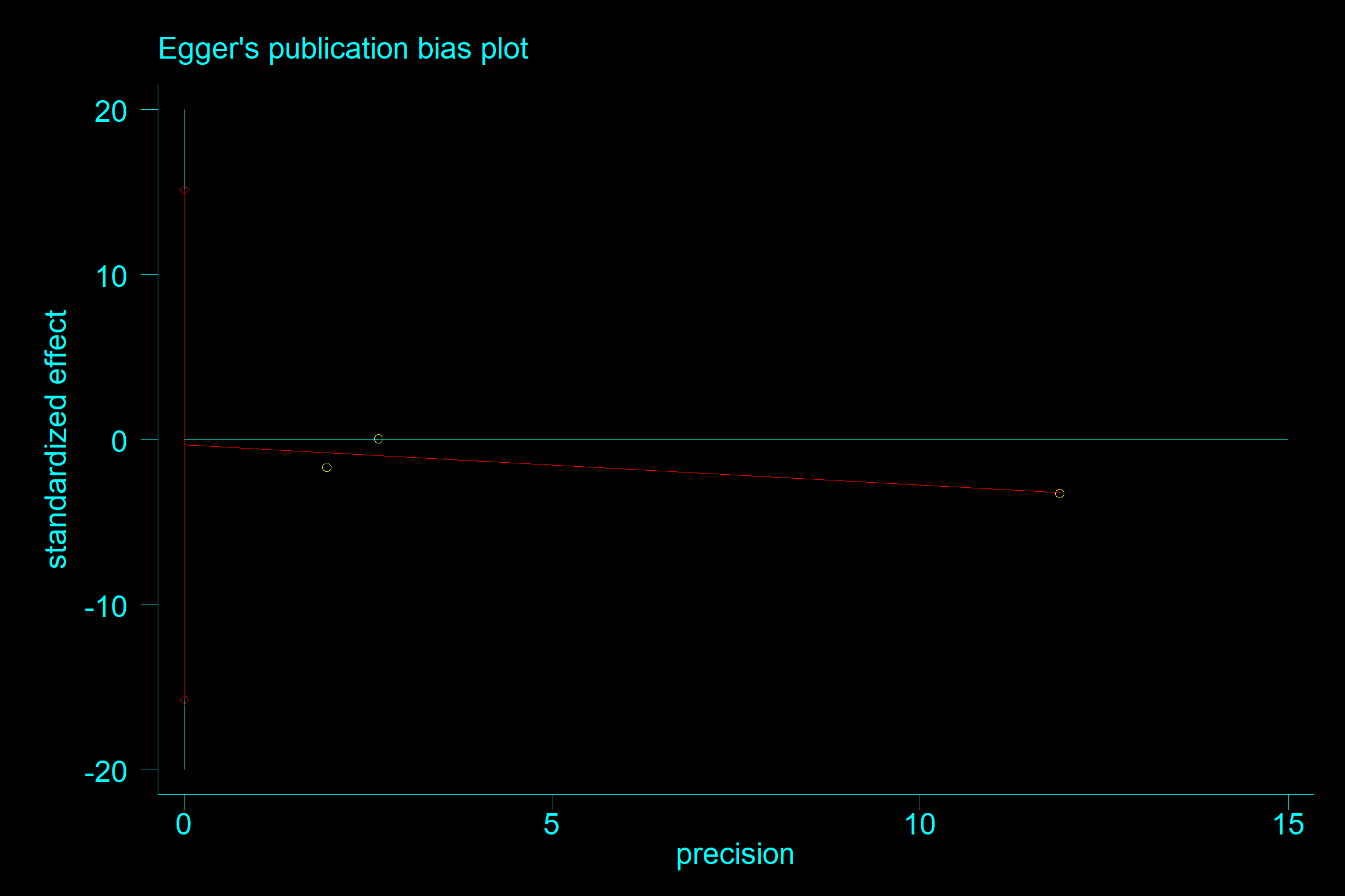


Supplementary Figure 9 Egger's funnel plot of HSD17B13 rs72613567: TA allelic variant in HCC patients compared with chronic liver disease (additive model).


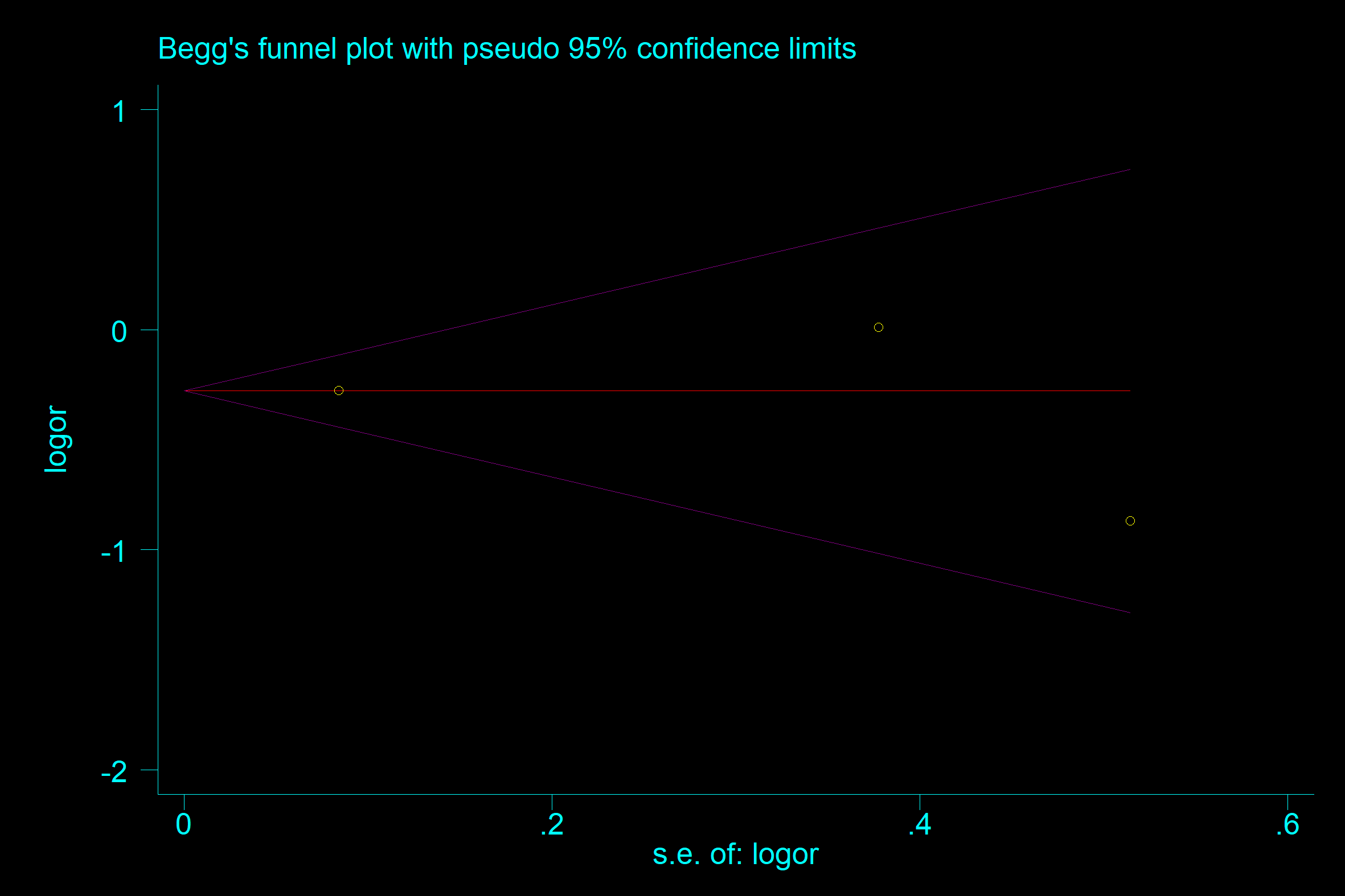


Supplementary Figure 10 Begger's funnel plot of HSD17B13 rs72613567: TA allelic variant in HCC patients compared with chronic liver disease (additive model).


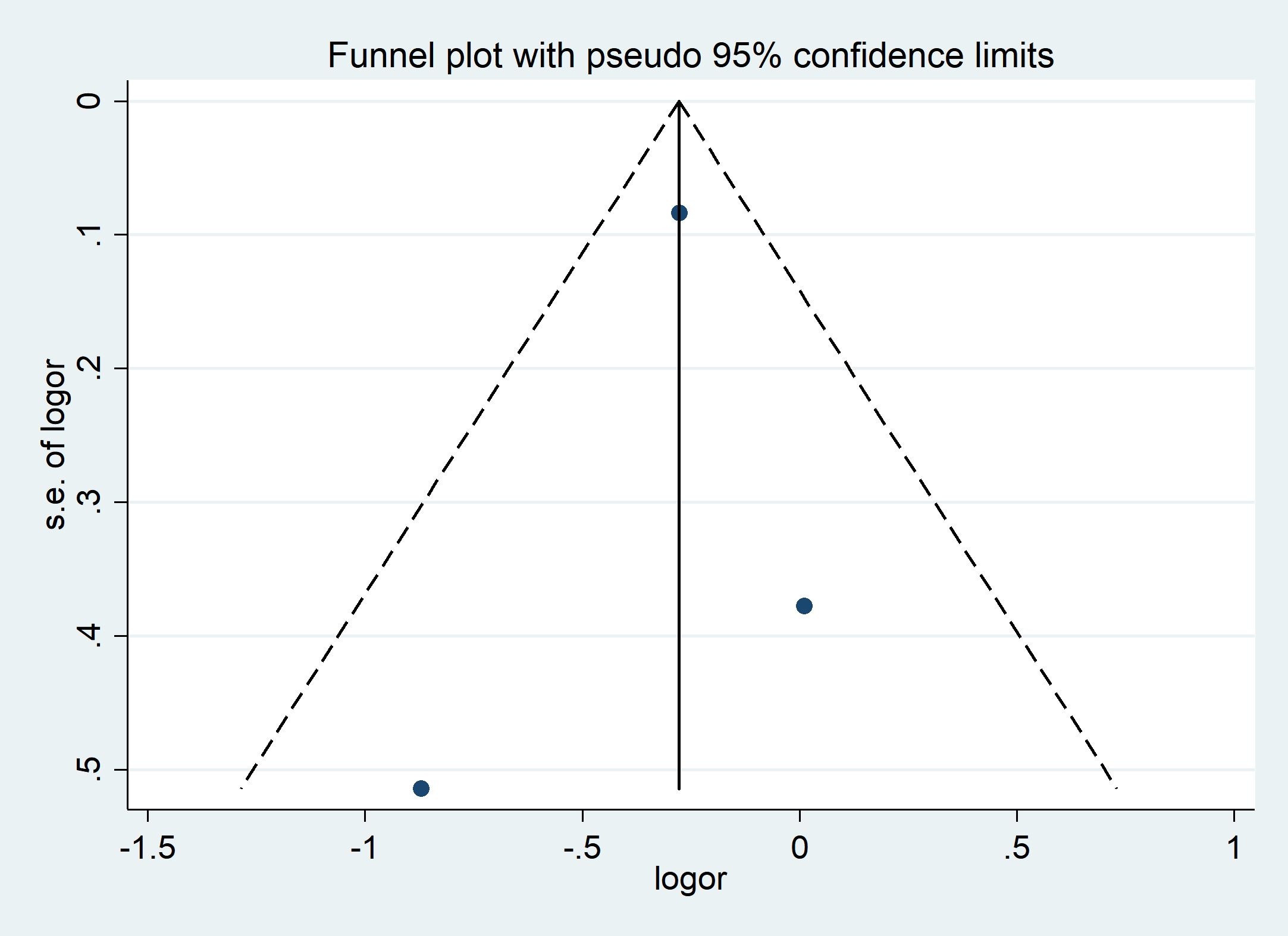


Supplementary Figure 11 Funnel plot of HSD17B13 rs72613567: TA allelic variant in HCC patients compared with chronic liver disease (additive model).


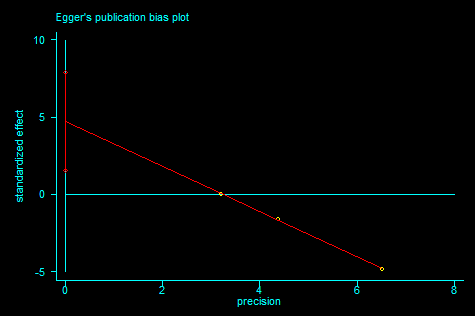


Supplementary Figure 12 Egger's funnel plot of HSD17B13 rs72613567: TA allelic variant in HCC patients compared with healthy controls.


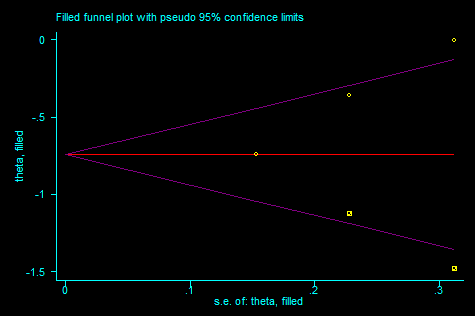


Supplementary Figure 13 Pruning method plot of HSD17B13 rs72613567: TA allelic variant in HCC patients compared with healthy controls.


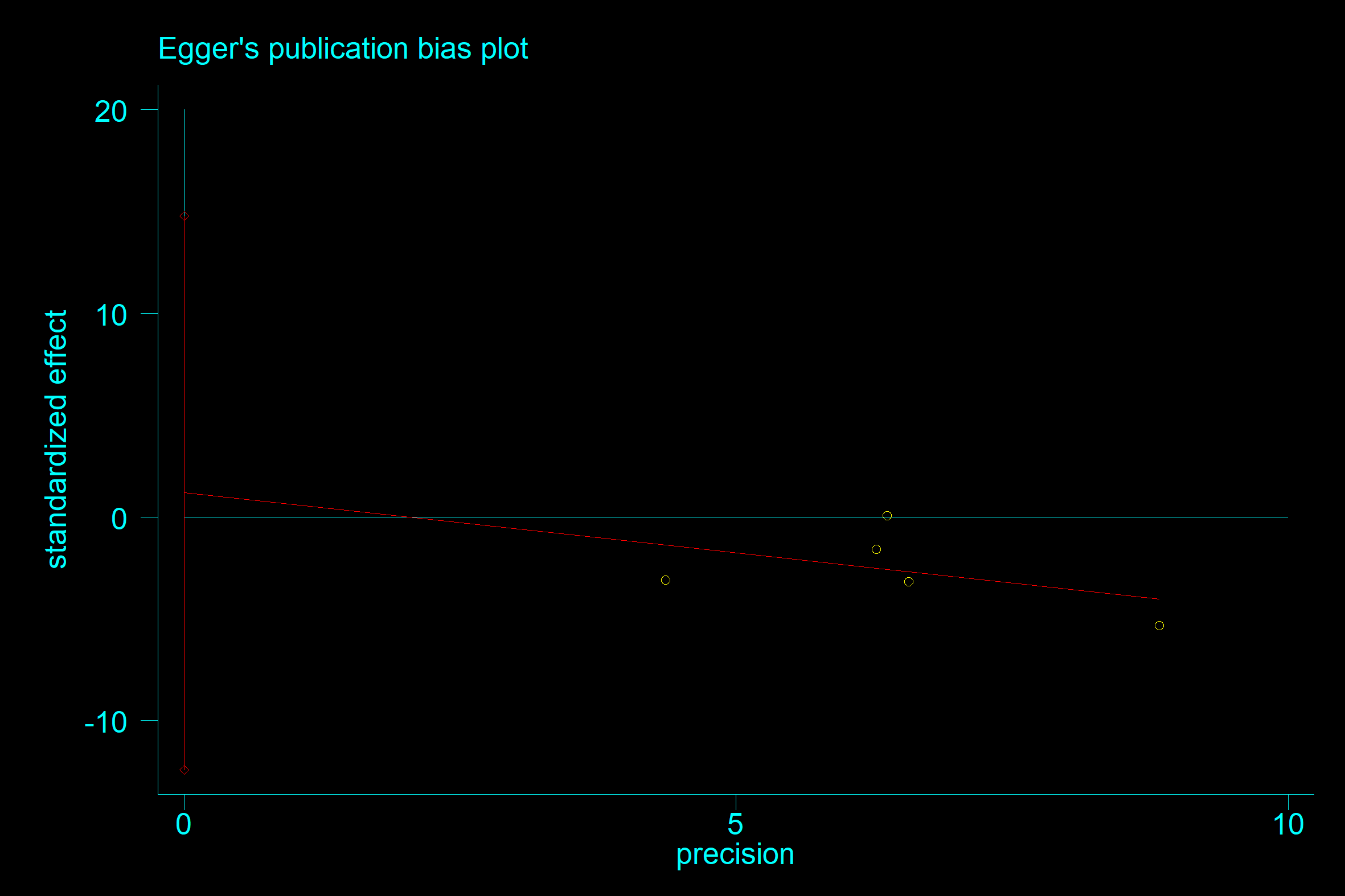


Supplementary Figure 14 Egger's funnel plot of HSD17B13 rs72613567: TA allelic variant in NAFLD patients compared with non-NAFLD.


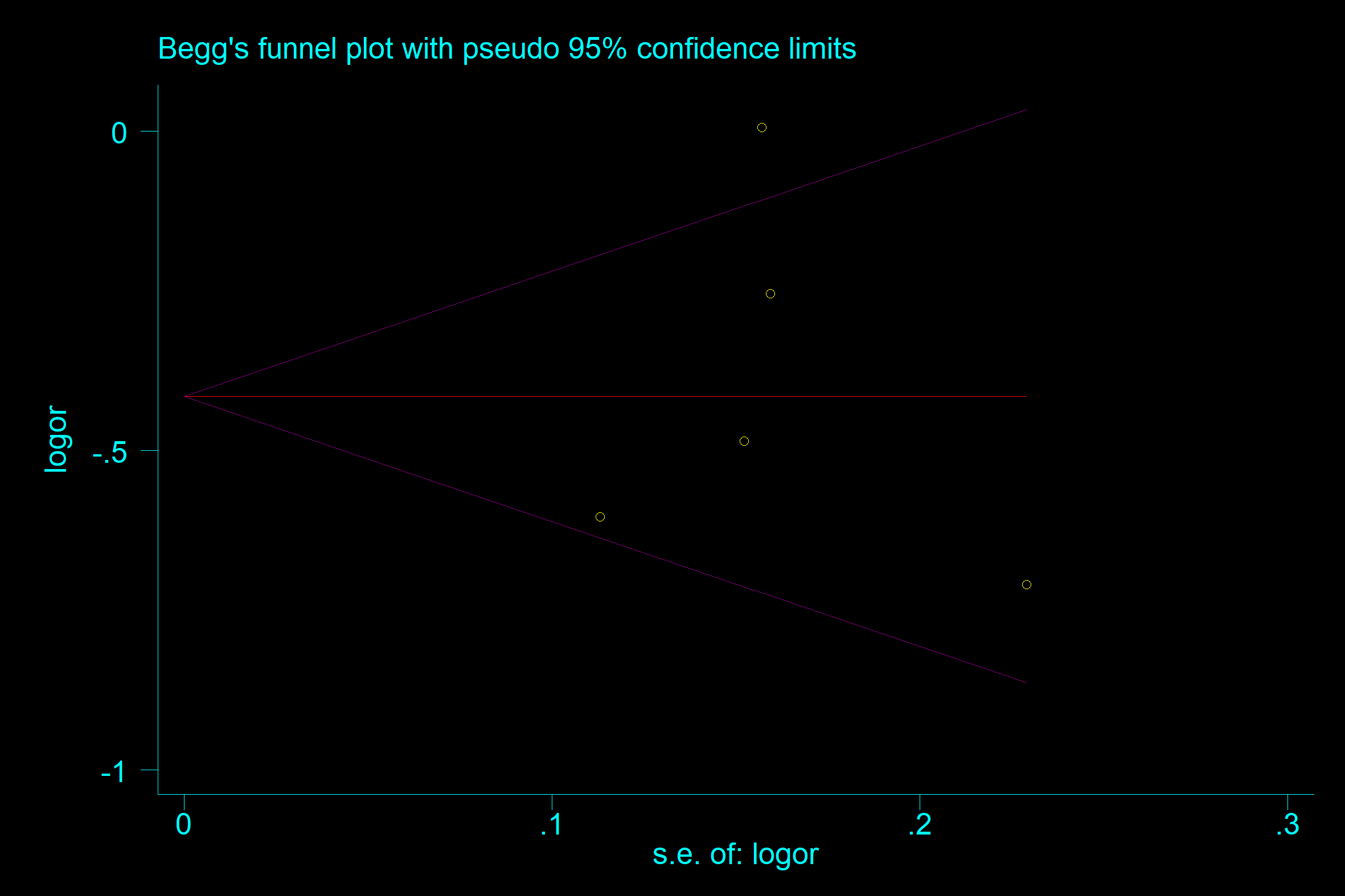


Supplementary Figure 15 Begger's funnel plot of HSD17B13 rs72613567: TA allelic variant in NAFLD patients compared with non-NAFLD.


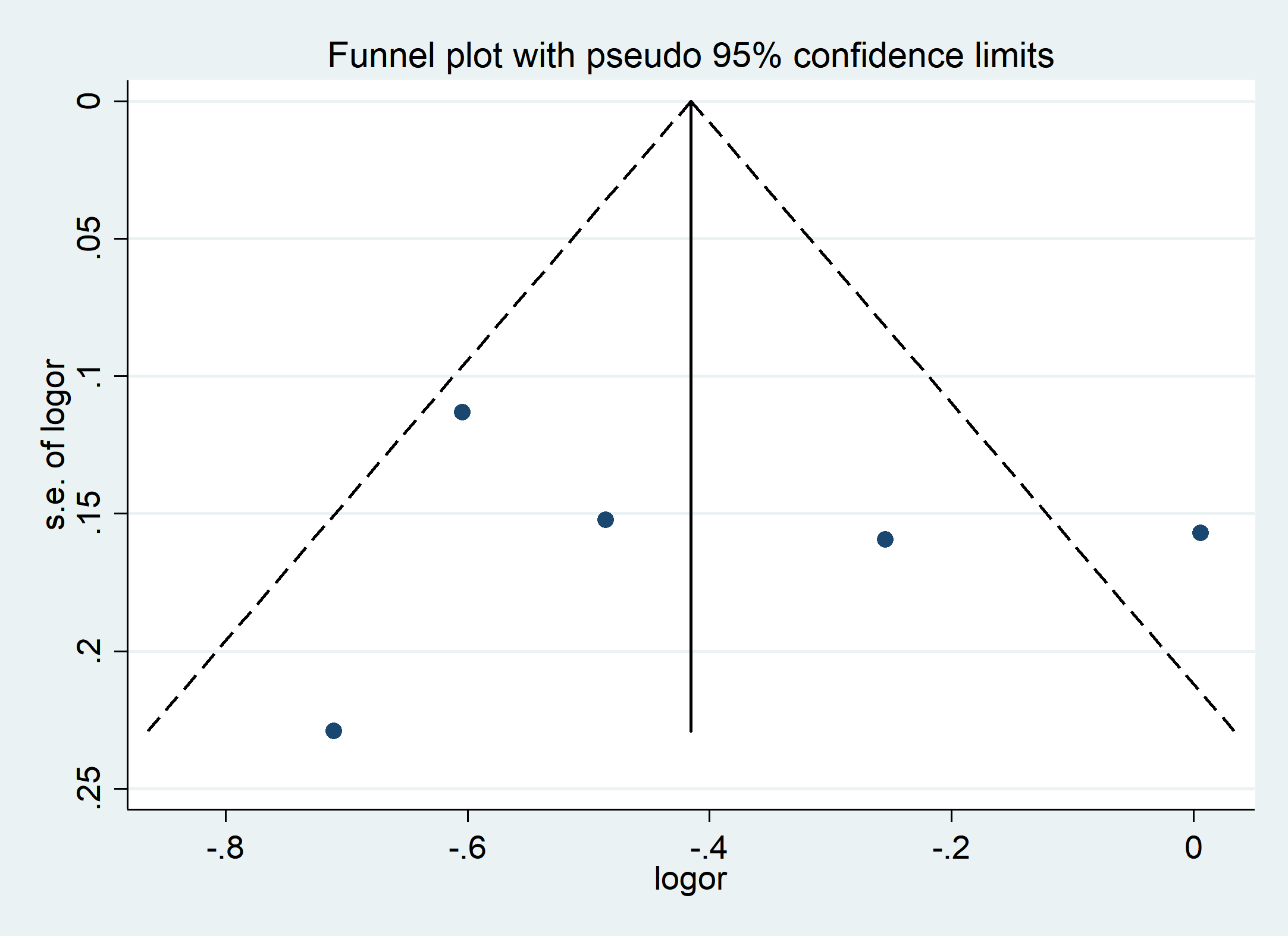


Supplementary Figure 16 Funnel plot of HSD17B13 rs72613567: TA allelic variant in NAFLD patients compared with non-NAFLD.


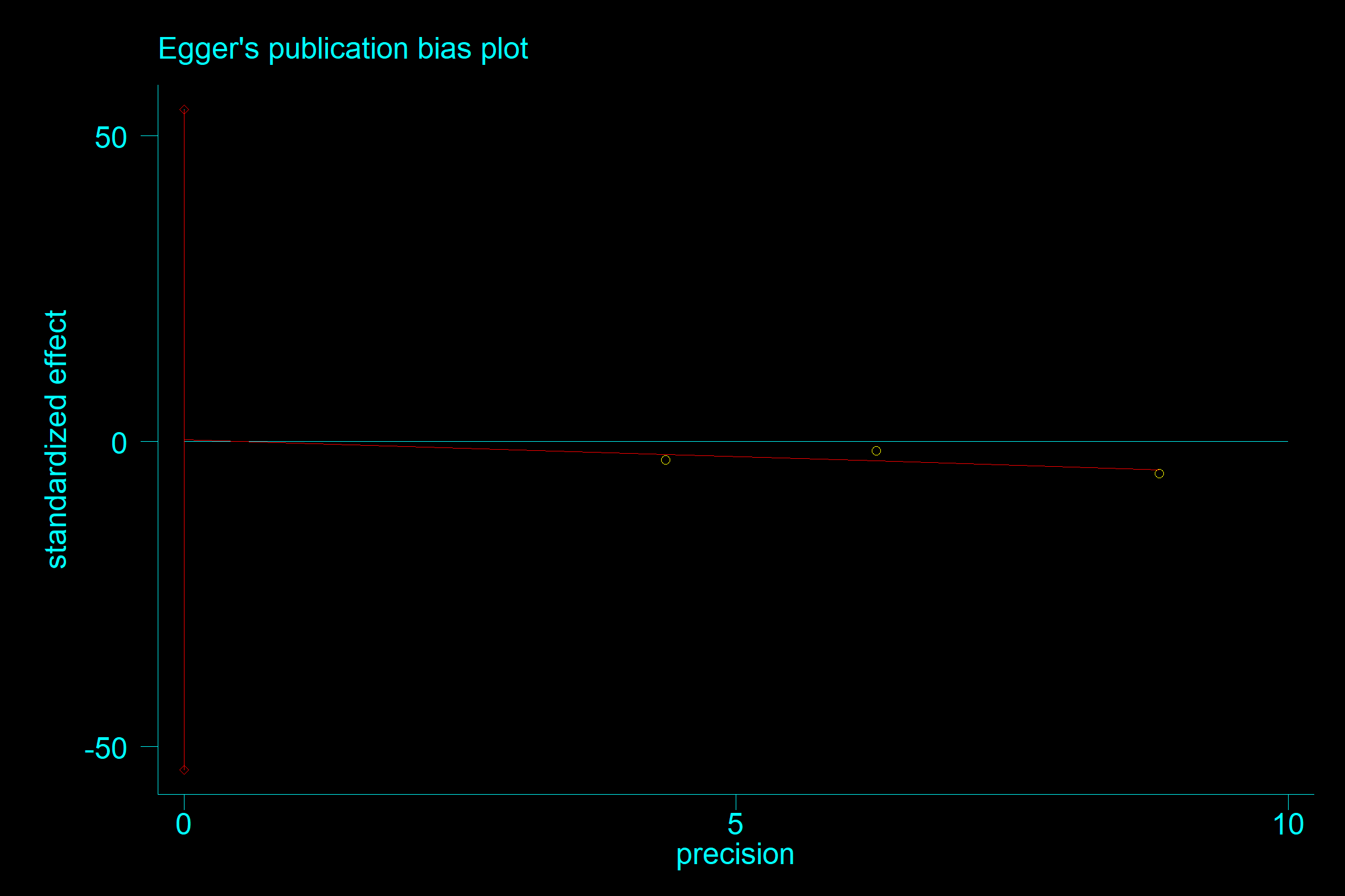


Supplementary Figure 17 Egger's funnel plot of HSD17B13 rs72613567: TA allelic variant in NAFLD patients compared with healthy controls.


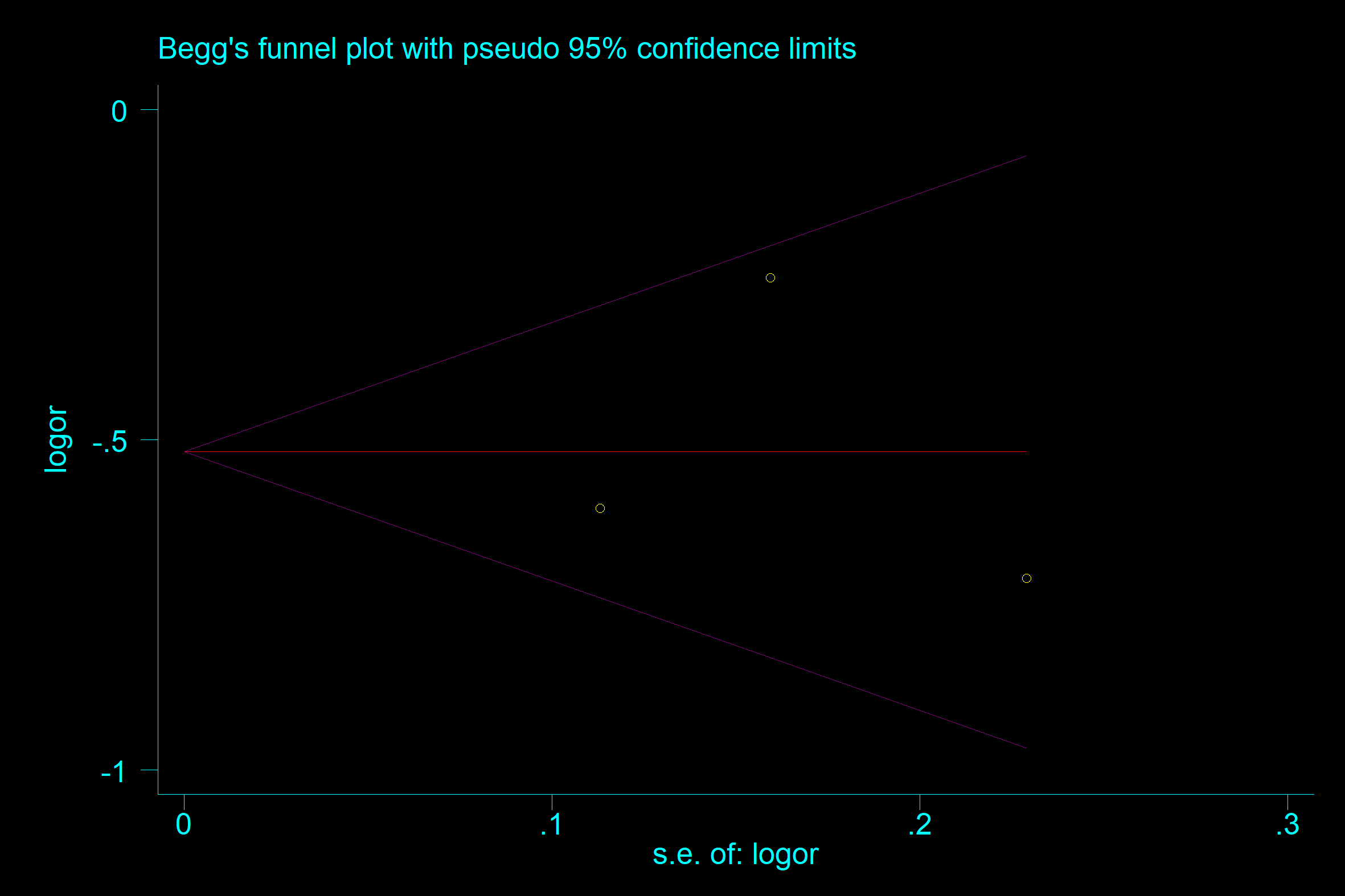


Supplementary Figure 18 Begger's funnel plot of HSD17B13 rs72613567: TA allelic variant in NAFLD patients compared with healthy controls.


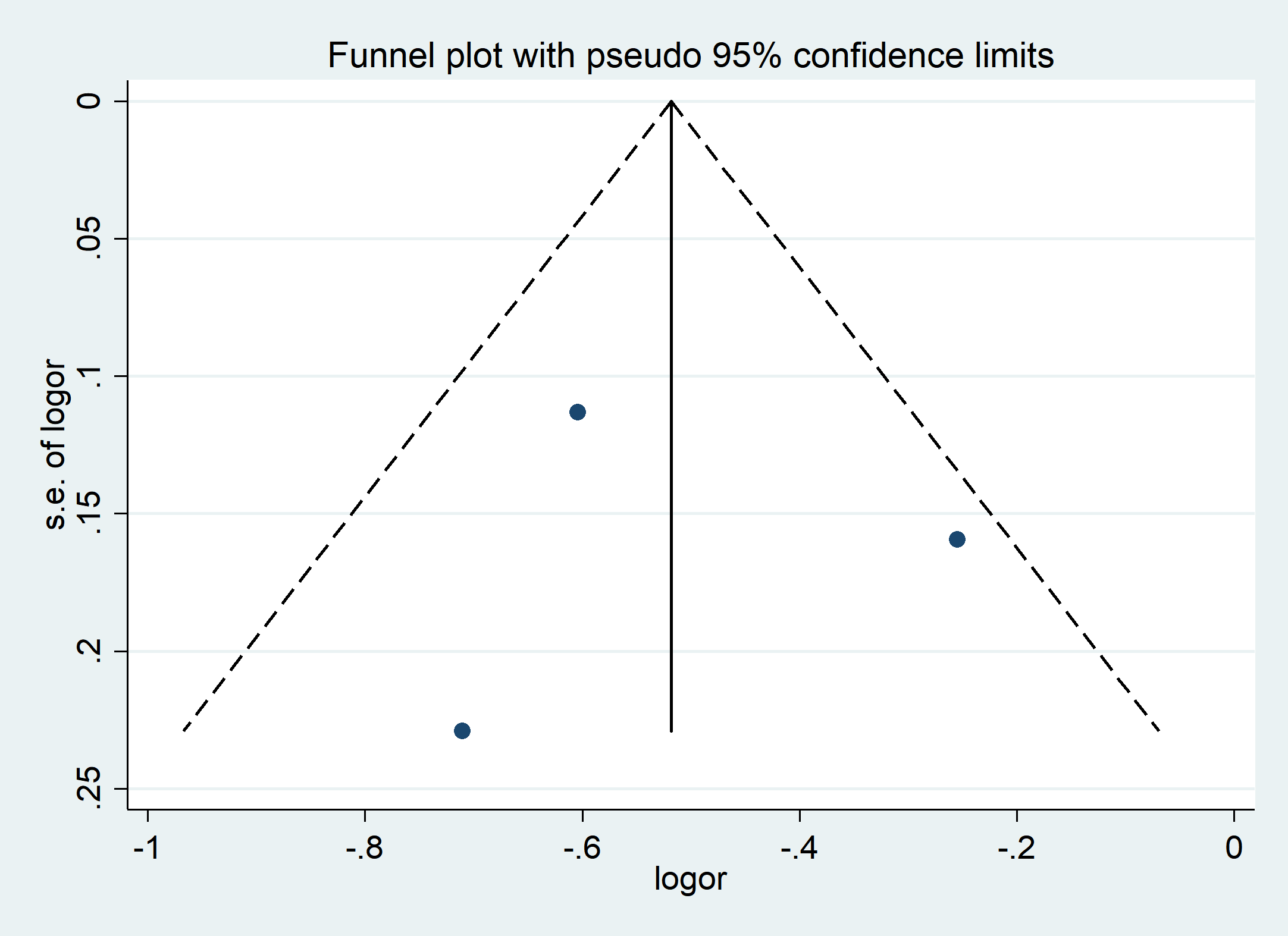


Supplementary Figure 19 Funnel plot of HSD17B13 rs72613567: TA allelic variant in NAFLD patients compared with healthy controls.
